# Smelly primes – when olfactory primes do or do not work

**DOI:** 10.3389/fpsyg.2014.00096

**Published:** 2014-02-12

**Authors:** M. A. M. Smeets, G. B. Dijksterhuis

**Affiliations:** ^1^Unilever R&D, VlaardingenNetherlands; ^2^Faculty of Social and Behavioural Sciences, Utrecht UniversityUtrecht, Netherlands; ^3^Section for Sensory and Consumer Science, Faculty of Science (FOOD), University of CopenhagenCopenhagen, Denmark

**Keywords:** olfaction, priming, cognition and emotion, behavior, valence

## Abstract

In applied olfactory cognition the effects that olfactory stimulation can have on (human) behavior are investigated. To enable an efficient application of olfactory stimuli a model of how they may lead to a change in behavior is proposed. To this end we use the concept of olfactory priming. Olfactory priming may prompt a special view on priming as the olfactory sense has some unique properties which make odors special types of primes. Examples of such properties are the ability of odors to influence our behavior outside of awareness, to lead to strong affective evaluations, to evoke specific memories, and to associate easily and quickly to other environmental stimuli. Opportunities and limitations for using odors as primes are related to these properties, and alternative explanations for reported findings are offered. Implications for olfactory semantic, construal, behavior and goal priming are given based on a brief overview of the priming literature from social psychology and from olfactory perception science. We end by formulating recommendations and ideas for a future research agenda and applications for olfactory priming.

## INTRODUCTION

There is a substantial literature from social cognition on priming, demonstrating the sometimes substantial and unexpected effects that environmental stimuli can have on information processing and behavior. Due to the traditional emphasis in psychological science on visual and auditory perception and language, only few priming studies employ olfactory primes. In contrast, there is a bountiful literature from the chemical senses community on the effects of olfactory stimuli on perceptual and cognitive processing that could be conceived of as priming research, but is not always discussed within a priming framework. These literatures seem somehow disconnected. In this review we intend to forge a connection between the two in order to explore how conceiving of odors as primes can help us make better sense of their potential for influencing human information processing and behavior. Secondly, we propose guidelines for how odors are best used as primes based on the intrinsic and sometimes unique properties of the olfactory system that can be seen as opportunities but also as limitations. More systematic research on odor priming could be envisioned to realize its full potential for applications if both properties and limitations are taken into account. We will formulate a possible research agenda for such research.

We will start by addressing what we actually mean by priming and primes.

## PRIMING IN THE SOCIAL SCIENCES

*Priming* refers to the phenomenon that incidental stimuli have been shown to influence higher-order cognitive and behavioral outcomes without the individual’s awareness or appreciation of this influence (Bargh et al., 2010). Interestingly, such “incidental” priming stimuli can be manipulated in the context of experimental studies to achieve effects in participants in a mere passive, inactive manner. This is opposite to earlier (social) cognitive approaches in which experimental manipulations used to be brought to the conscious attention of participants to study how they affected decision-making (Bargh, 2006). To illustrate the former, Bargh (2006) gives the example of how polite behavior can be studied in an experiment in which the concept of politeness is passively manipulated by embedding adjectives related to politeness in a scrambled word test disguised as a language test, which is then followed by an opportunity to behave politely. Thus, priming research allows us to investigate how higher mental processes such as judgment and social behavior can be triggered and then operate in the absence of conscious awareness (Bargh and Morsella, 2008).

The notion that environmental stimuli can prime behavior is interesting, as it implies that there is a bridge between perception of the stimulus (e.g., a *word *related to politeness) and motor behavior (the polite *behavior* of waiting for someone to finish speaking instead of interrupting) possibly in the form of an activated mental *concept* of politeness. Specifically, by presenting words or images, the underlying related *concept* becomes accessible – an associative process – for further information processing (cf. Loersch and Payne, 2011). The mental content that has thus become available is now likely to be used as a source of information in subsequent information processing and behavior. Loersch and Payne (2011) distinguish between four types of priming: semantic priming (category identification), construal priming (judgment), behavior priming (action), and goal priming (motivation). Whichever type of priming occurs depends on whether the current situation invites, e.g., judgment rather than behavior or vice versa, and on other attributes such as a person’s attitudes toward a primed category, personal goals and interests or constraints of the situation. A good example of the latter is an experiment by Cesario et al. (2010) in which participants who were in a enclosed booth when primed with a social stereotype of aggression, chose for a fight-like behavioral response, whereas those who were seated in an open field chose for a flight-like behavioral response.

An important consequence of Loersch and Payne’s situated inference model of priming is that single primes can have multiple effects (i.e., as either one or more types of priming), but also that these effects are not the same for everybody or in all situations. This has special relevance for applications of priming in the real world, where individuals and situations will differ greatly.

In addition to the four types of priming that are central to the Loersch and Payne framework, we can distinguish between *perceptual* priming, *repetition* priming, and *affective* priming which bear relevance to odor priming. We speak of perceptual priming when prime and target share perceptual attributes. This is not the same as semantic priming. For example, Koenig et al. (2000) found that while odors presented during a learning phase acted as perceptual primes when participants were presented with these odors again during a test phase, there was no such priming effect when odor names – rather than odors themselves – had been used during the learning phase. The explanation for the difference may lie in the fact that perceptual priming involves modality-specific subsystems in memory, whereas semantic priming involves associative (amodal) subsystems in memory (Koenig et al., 2000). Repetition priming refers to the phenomenon that a stimulus can act as its own prime. When presented again, an odor is processed faster because its representation in memory was activated just before, and there is still a memory trace available. For odors, this was discussed by Olsson et al. (2002). They conclude that in some of the older repetition priming literature it is hard to disentangle purely olfactory priming from semantic priming – which is related to the previously mentioned distinction between perceptual priming and semantic priming. In Olsson (1999) even negative priming occurred when odors that were correctly identified were proven to be processed more slowly than odors that had not been identified. Identification of odors allowed for verbal labeling and may have led to semantic overshadowing (cf. Melcher and Schooler, 1996). Finally, in affective priming there is an unintentional influence of a first evaluative (affective) response, acting as a prime, on the subsequent processing of a target stimulus. For example, the positive affective tone of primes (often words) may activate affectively congruent material in memory (Klauer, 1997). Explanations have been sought in affective congruency between prime and target (both “positive” or “negative”), but also in congruency in response tendency. Consequently, positive affective primes would facilitate (congruent) approach responses, and negative affective primes would facilitate (congruent) avoidance responses to affectively congruent targets (Förster and Liberman, 2007). Odors may be potent affective primes as will be highlighted later in Section “Priming via valence”.

Central to many explanations of *how* activated concepts can prime behaviors is William James’ *ideo-motor* *action* principle which holds that activation of a cognitive representation of an action increases the likelihood of that action being carried out, via the triggering of active behavior representations, which cause movement of relevant muscles (Schröder and Thagard, 2013). Deliberate choice or motivation is not considered to be necessary. Priming effects, then, occur as a result of the *spreading of activation*, by which activation of one node in memory automatically spreads to another. Thus, priming effects are effortless and uncontrollable. For a more detailed account of how this might work involving computational modeling and neural networks, the reader is referred to Schröder and Thagard (2013).

Priming effects are supposed to take place outside of awareness. Social and cognitive psychologists have somewhat different perspectives on this. In cognitive psychology, awareness in this context would be equated with ability to perceive. For example, individuals could only be presumed to be unaware of a stimulus if stimulus intensity or duration would be below perceptual threshold (hence, at subliminal levels). According to Bargh (1992), it does not matter much from a social psychologist perspective whether someone is aware of the stimulus event, as long as the individual remains unaware of the ways in which the stimulus is interpreted and of the influence of this awareness on subsequent processing. Both subliminal as well as supraliminal primes have been proven to be effective primes (Bargh and Morsella, 2008). Goal or need state play an important role: for example Karremans et al. (2006) demonstrated that subliminal priming with a brand drink name such as Lipton Ice Tea positively affected participants’ choice for and intention to drink the primed drink, but only for those who were thirsty.

To conclude, subliminality of stimulation could be important but only because if the individual is unaware of the stimulus event we can be sure they are unaware of the potential influence it has on their behavior. And, even when people are able of perceiving a priming stimulus, we might still conclude its subsequent effects on behavior take place outside of awareness.

So far we have seen that priming refers to the ability of “incidental” environmental stimuli to influence higher order cognitive processing and behavioral outcomes, and that these influences occur outside of awareness, effortlessly, and automatically. Mental representation of concepts play an important role, as activation of such a concept by a prime can lead to the simultaneous triggering of other cognitive, motivational, and behavioral processes by spreading of activation in memory. Both supraliminal as well as subliminal stimuli have been shown to be effective primes. Before we continue to look at the suitability and effectivity of odors as primes, we will first explore the unique properties of the sense of smell.

## UNIQUE PROPERTIES OF OLFACTION

We are about to make claims about the suitability of contextual odors as primes. We start by introducing an important distinction: that between *odor* and *odorant*. The term *odorant* refers to the volatile chemical substance that is capable of eliciting the experience of an *odor* – it can be a single compound as well as a mixture consisting of a large number of compounds. The *odor *exclusively refers to an individual’s experience, it is a percept. The olfactory experience (an “odor”) is in all likelihood elicited by an *odorant*, but there have been occasions in which odor experiences have been reported even in the absence of an odorant. In a study by Knasko et al. (1990) the presence of an odor was strongly suggested by the context. Participants who were given the suggestion of a pleasant odor being in the room reported a more positive mood. A more extreme example is reported by O’Mahony (1978) who told a compelling story on TV which resulted in people calling the TV station stating that they had indeed smelled an odor emanating from their TV set. Furthermore, it is possible that a certain odor, experienced as resulting from a specific odorant, is not experienced by 100% of the subjects. Some subjects may perceive another odor, based on, for example, prior (lack of) experience with the odor. 

Odors in memory are also referred to as *odor objects*, that, even when consisting of ten or hundreds of volatile components (the *odorant*) are perceived as unitary perceptual events (the *odors*) against a continually shifting olfactory background (Stevenson and Wilson, 2007). This goes to illustrate that there is not a necessary relation between the chemical properties, or even the presence, of an odorant, and the odor perceived as resulting from it (cf. Wilson and Stevenson, 2006). A focus on the so called “stimulus problem” (Stevenson and Boakes, 2003) will likely lead to incomplete theories and remain insufficient to understand olfactory perception in its entirety.

### SENSE OF SMELL IS AN IMPLICIT SENSE

The sense of smell has also been alluded to as a hidden or *implicit *sense (Köster, 2002). Because vision is usually in the center of our attention, it is presumed to be the dominant sense, followed by the senses of hearing and touch. As a result, people tend to be less aware of odorants in their environment. Odorants, after all, cannot be seen or heard, and they can only be felt if they are at high enough concentrations to stimulate the trigeminal nerve innervating the nose, throat, mouth, and eyes, which induces sensations of tingling, prickling, burning, or even pain (Doty et al., 2004). There are large individual differences in the tendency to be aware of odors such that some people never seem to notice any, and would go to sleep without problem on a mattress on which the cat had just peed, whereas others are quick to notice any unpleasant or pleasant odors and would avoid them or seek them out purposefully (Smeets et al., 2008). Regardless, odorants –and their odors – are in general unlikely to draw attention unless they are especially pleasant, overly strong or an assault to the senses, or if they signal danger (fire, gas leak) or contamination (rotten foods, cadavers). According to Stevenson (2010) these are events which we have been “programmed” to attend to and as a result related approach or avoid-behaviors are hard-wired in the brain. Odors can also draw attention if they are especially meaningful to a person, i.e., they are learned to carry significance, be they approach or avoidance triggering (e.g., the perfume of your ex who left you).

There are several factors possibly contributing to odors taking a backseat among our sensory systems. One is that odorants spread, become diluted and are hard to pinpoint to a particular source. Thus adapted odorants cannot be easily localized and form a background for novel odor objects to figure against (Stevenson and Wilson, 2007).

With relevance to olfactory priming, odors appear to be perceived under different awareness circumstances:

1. Attentively: identifiable using verbal label: “I smell banana,” or not identifiable: “I smell something, but I don’t know what it is.”
2. Semi-attentively: noticing there is something special, but not being able to pinpoint it (e.g., when one notices there’s something different about a colleague –“Do you have new glasses?” and finding out he grew a mustache). With ambient odors this could be: “There’s something special with this room today, but I can’t really tell what it is.”
3. Inattentively: subjects show no evidence of being aware of something in particular (“…”).

### ODORS QUICKLY ADAPT

Odor receptors are *quick to adapt*, Adaptation here refers to the “waning of response with stimulus repetition” (Dalton, 2000, p. 488) often referring to peripheral and physiological sensory processes, though “central adaptation,” occurring in higher nervous centers can occur too (habituation). In olfaction peripheral adaptation is the much more common and stronger process. The adaptation process typically leads to a reduction in perceived intensity which can occur with even a few breaths of air containing an odorant (Dalton, 2000). The advantage of adaptation lies in a flexibility of the system to quickly tune into change. So, by current odor experiences merging with the background, chances of detecting novel odorants (e.g., by their odor) are much enhanced. The fact that olfactory adaptation is quick to set in, does not mean the olfactory stimulus ceases to have an effect on information processing after its onset. We recently observed effects of being exposed to sweat odor on facial emotional expressions (in this case fear and disgust) measured using facial EMG-electrodes lasting for at least 6 min, which is well beyond the time in which adaptation to the smell would have occurred (De Groot et al., 2012). The perception of the odor may have set in motion other processes that persist even after olfactory adaptation has set in.

So, to summarize, with one of the requirements for successful priming being that the individual is unaware of priming effects, the fact that humans are hardly aware of odors at all, and are quick to adapt to the sensation, makes odors good candidates for effective priming.

### ODORS ARE STRONG TRIGGERS OF EMOTIONAL MEMORY

Another interesting characteristic of odors is that they are *strong triggers of emotions*. This is also known as the Proust effect, referring to the experience described by Marcel Proust in *A la Recherche du Temps Perdu* (Proust, 1913 in Jellinek, 2004) of his protagonist Swann feeling overcome with melancholy and emotion when experiencing the smell (and taste) from biting into a Madeleine after dipping it into tea. Inspired by this phenomenon many scientists devoted themselves to answering the question of whether odors are in fact stronger triggers of emotional memories than perception in other modalities. Note that Proust needed several pages to describe the mental search before finding the reason for the emotion. This illustrates the fact that the link between the prime and its effect normally escapes awareness. While the final judgment is still out on this (Jellinek, 2004; Gilbert, 2008; Toffolo et al., 2012), the ability of odors to trigger emotions make them suitable *affective *primes.

### HUMAN ODOR CATEGORIZATION AND IDENTIFICATION: AMATEUR AT BEST

The connection between odors and language is a problematic one. Most individuals experience *odor-naming problems*. Even when an odor is common or seems very familiar, verbalizing it can be difficult (Cain, 1979); the tip-of-the-nose phenomenon (Lawless, 1977). People can classify odors into categories such as fruity, floral, and putrid, but there is little consensus on what the basic categories are (Wise et al., 2000; Auffarth, 2013), or even if they meaningfully exist. Categorization would be based on coarse perceptual features with boundaries between categories being rather fluid.

This problem with verbalizing odors may be related to the poor relation between the piriform cortex in which odor objects and categories are encoded, and the language network, e.g., cortical areas mediating odor naming and identification (Olofsson et al., 2013). It could be that from an evolutionary point of view, naming odors was never very important – performing immediate motor-induced actions either to approach or avoid was. Based on research in patients with semantic primary progressive aphasia, who suffer from extensive temporal lobe atrophy, Oloffson et al. posited that odor object information – even in healthy humans- is still relatively coarse and unprocessed compared to visual object information by the time it arrives at the lexical-semantic network in the brain. This would be due to fewer unimodal areas available for object processing in the olfactory than in the visual system prior to its arrival at the lexical-semantic network via the temporal lobe which constitutes a bridge into this network. The results may be mapping imprecision and object mismatch of odor objects. The authors conclude that because of this, odor object identification is more vulnerable to perceptual ambiguity. This phenomenon might have serious consequences for odor priming, as it casts into question the very ability of odors to link into specific, and unified, concepts which is central to conceptual (semantic) priming.

### MOST IMPORTANT DIMENSIONS OF ODOR INFORMATION PROCESSING

Valence – varying from unpleasant to pleasant – is considered to be the most important odor dimension (Engen, 1982; Kaeppler and Mueller, 2013). Other dimensions considered as primary and employed in many studies (Kermen et al., 2011; Kaeppler and Mueller, 2013) are intensity, edibility and familiarity. Classifications of odors, relying on approaches asking subjects to engage in sorting, similarity judgments or sensory profiling have not led to universally agreed odor classes (Wise et al., 2000). One of the problems is that untrained subjects have great difficulty disregarding the valence dimension when asked to rate or classify odors (Kaeppler and Mueller, 2013) which in fact confirms its primacy in odor judgment. The dimensions of valence, intensity, edibility and familiarity seem to support the three major functions of olfaction as distinguished by Stevenson (2010), with digestion (i.e., appetite regulation) as first function being followed by avoiding environmental hazards (such as fires or rotten food) and social communication. All three functions, from an evolutionary perspective, subserve approach and avoidance behavior aimed at enhancing an individual’s chance of survival.

### ODOR PRIMING: WHAT’S DIFFERENT?

As previously noted, priming relies in large part on improving accessibility of conceptual representations for further information processing as a result of “incidental” perceptual stimulation. The question lying before us is whether priming with odors is in any way different from priming with visual stimulation in the form of images or words? We will reflect on the four types of priming distinguished by Loersch and Payne (2011): semantic priming (category identification), construal priming (judgment), behavior priming (action), and goal priming (motivation), and because of its special relevance here, to affective priming.

### OBSERVATIONS ON BEHAVIORAL AND GOAL PRIMING WITH ODORS

When it comes to action priming or motivation priming, it seems obvious that odors are just as potent as (and sometimes even more potent than) visual stimuli. For example, immediately removing oneself from dangerous situations (a fire or a gas leak) or rotten food is a behavioral response that is in the interest of avoiding environmental hazards [Stevenson’s (2010) second function of olfaction] and which relates to the primary dimensions of odor such as valence, edibility and familiarity. Although such reactions may not qualify as primes when individuals are aware of the link between the odor and the emotional (fear or disgust) and behavioral (moving away from the source) response, they are very much automatic. In a classical conditioning study, the low-level and briefly presented unpleasant odors of “rotten egg” and “sweaty socks” were successfully employed as aversive unconditioned stimuli to change expectations to a conditioned stimulus in the form of a human face (Gottfried and Dolan, 2004). This demonstrates how salience and automaticity of odor stimuli can affect information processing even when awareness of the odor must have been low. Semantic processing of the stimulus need not necessarily be invoked to yield action effects.

Odors are also effective as *goal *primes. Delicious food odors, in line with Stevenson’s first function of olfaction – digestion and appetite regulation – may subconsciously divert a person from pursuing an ongoing goal and tempt people to start eating. Food courts in airports tend to have these effects and food odors – typically freshly baked bread – deliberately spread in supermarkets could lead to purchasing behavior. The first author, who on her daily train travel to work passes by a coffee factory spreading coffee roasting odors, has often observed other passengers formulating a desire for coffee or concrete plans to purchase some at the next station. In Gaillet et al. (2013) the odor of melon or of pear was unobtrusively presented. In a later choice test the group exposed to the melon odor chose more starter items consisting of fruit and vegetables, and the other (pear) group chose more desserts with fruit. Only the melon group had shown a decreased reaction time in a Lexical Decision Task for the word “melon.” The effect on menu choice can be seen as goal priming, where melon – a typical starter item in France, the country of the study – led to an increase in choices for fruit/vegetable starters, and pear – a typical dessert item – led to an increase of choices of fruit desserts. Obviously we see here an odor priming effect, but rather than to conclude that it involved a semantically mediated concept of “pear” (which did occur for “melon”), both odors resulted in effective goal priming.

In a different domain, Miller and Maner (2011) showed that scent cues associated with female fertility (T-shirts worn by women in the late follicular versus luteal phase) enhanced reported perceptions of womens’ sexual arousal in odor-sensitive males which the authors interpreted in the context of goal pursuit. The major dimensions of odor perception, valence, and edibility, can facilitate goal priming responses, again without semantic processing being required. It is thus safe to conclude that odors make for very effective behavioral and goal primes.

### OBSERVATIONS ON SEMANTIC AND CONSTRUAL PRIMING WITH ODORS

Semantic and construal priming via odors, in view of the specific characteristics of odor just listed, is more complicated. Can the odor of camembert cheese, for example, prime words related to other typically French food products or a typically French sports event such as the Tour de France? Since we have seen that categorization and identification of odors is problematic, and since the semantic route partially relies on, or most certainly benefits from, such processes, semantic odor priming cannot simply be assumed to take place. For example, we might expect that *seeing* a picture of a camembert cheese likely activates mental representations of other foods (French) cheeses, other typical French food products such as baguettes, or even other French words, via conceptual links with the product, once recognized or identified. However, we cannot simply assume the odor of camembert to accomplish the same. We might expect the odor to be categorized as belonging to the food category, and even as cheese. Thus, via the semantic route, the odor of camembert might be a good prime for other food or cheese concepts (like beer can prime – a desire for – pretzels; Hyde and Witherly, 1993). However, many people would not be able to categorize the smell as (French) cheese or identify it as camembert. Due to the ambiguity inherent to the sense of smell, some might misconstrue it as body odor which would lead to another priming outcome altogether than would be the case if a visual prime of cheese had been used. Depending on the interpretation of the odor prime, the subsequent effects on judgment of an object or situation will be vastly different. Note that De Araujo et al. (2005) showed a difference in perceived valence depending on whether the odor was labeled as a body odor or as cheddar cheese.

This brings us to the fact that the characteristic odor of French cheeses can elicit *affective* reactions. The cheese odor represents an edible food product that is liked or disliked. As valence may be the primary dimension along which priming occurs in this camembert example, individuals may now have easier mental access to other well-liked or disliked products, or may show behavioral responses of approach of avoidance, respectively. But: would we expect the *odor* of camembert to cause shorter reaction times to French words – as, e.g., measured with a Lexical Decision Task – than to other language words in a priming task, which we would if *pictures *of camembert were used as primes? Probably not. Instead, priming with camembert *odor* might enhance the mental processing of words of other *liked* food products (or liked products in general) in a cheese-lover, create an approach response toward anything that follows such priming, or enhance the possibility for positive construal of an object, person, or situation.

Aside from a valence-route for priming with camembert odor, an individual who once enjoyed camembert with friends during a wonderful vacation in France might re-experience that memory and find themselves taken back there. Now priming may occur based on the content of the *autobiographical memory*. Thinking back of how lovely the French countryside was, would cause a spreading of activation to the concepts “France,” “countryside,” and “French countryside.” A subject might now show a fast response to words related to the Tour de France on this basis, as it is now the memory providing the link to language and visual mental representations. Likewise, they may show speeded recognition of contextual features such as red-white checkered tablecloths because such a tablecloth happened to be part of their memory. Someone who did not have such a memory, would not show such a response, which makes this response differ strongly across individuals and almost impossible to systematically investigate.

Finally, there is a possible priming route that involves *mood*. This is in line with literature demonstrating that mood at the time of judgment can be used as information by an individual to reach a judgment, for example on how happy or satisfied one is (Schwarz and Clore, 1983, 2003). On a similar note, effects of odors on feelings of wellbeing and health in aromatherapy have been contributed to changes in mood caused by a strong liking for the odor (Stevenson and Boakes, 2003; Herz, 2009). This implies that the pleasant aromas do not directly reduce stress and increase relaxation via physiological changes induced by odorant inhalation. Rather, feelings of stress and relaxation are influenced in the same way as being in a good mood would, with the odor being the mood-enhancer. Liking camembert may put someone in a good mood when smelling it. Priming via mood might help explaining effects such as seen in the famous “the smell of helping” study by Baron (1997) in which it was demonstrated that passersby in a shopping mall were more inclined to help a same-sex accomplice (e.g., by picking up a dropped pen) when a pleasant ambient odor (e.g., of baked cookies) was present then in the absence of such an odor. Here, the odor of baked cookie primed the act of picking up a pen. It is unlikely that this involved a semantic route. After all, there is no clear conceptual relation between baked cookies and helping behavior. Would seeing pictures of cinnamon buns act as primes to helping others in the same way? It could if you really love cinnamon buns, and the mere sight of it improves your mood, but the odor may be a more direct route into mood and emotion, as previously argued. Thus, while semantic priming via odors can be problematic, effects that suggest semantic odor priming may be explained by alternative routes into the concept, such as via priming of memories that facilitate spreading of activation to any concepts related to that memory. As a consequence, substantial individual differences are expected, as autobiographical memories are unique. Likewise, odor primes intended to be semantic primes may inadvertently lead to affect (valence or mood) priming, thus yielding behavioral effects that never involved the underlying (semantic) concept. We would conclude that odors do not make for good semantic primes, but can nevertheless have effects that may be interpreted as such, by spreading of activation traveling via indirect “autobiographical” routes or via valence transfer, that eventually can be linked to semantic concepts indirectly. Of course, behavioral and goal priming could result via similar mechanisms.

## RECOMMENDATIONS AND APPLICATIONS FOR ODOR PRIMING

### PRIMING VIA VALENCE

One of the conclusions reached so far held that odors can make for good behavioral and goal primes along the primary dimension of valence. This is strongly related to the notion of *affective *priming, previously discussed in Section “Priming in the Social Sciences.” Affective priming phenomena may have an adaptive function, in that they serve to quickly serve opportunities and threats in the environment (Klauer, 1997). Odors, generally evaluated primarily in terms of affect (good or bad) may therefore constitute important affective primes. For reasons of ease of comprehension, we propose to include all influences of odor valence priming under the header of affective priming for our current purpose. This is irrespective of whether the affect association originated from the various types of learning involving odors (Stevenson and Boakes, 2003), or from congruent mood, and irrespective of the underlying mechanisms (e.g., congruency of stimulus and response). This type of affective priming is prevalent in applied settings such as stores, parking garages, public transportation, health care settings, the workplace, etc., where positively valenced odors have been dispersed to trigger approach behavior (consumption, purchase), positive feelings (safety), a sense of wellbeing, work engagement, and so on. A few comments are in order: in many cases strong odors are used. When odors are strong and easy to notice, their influences on human cognition and behavior cannot be classified as primes under the definition that requires effects to take place outside of awareness. Strong odors tend to be disliked by people who are sensitive to strong stimulations (Doty et al., 2004) or score high on the avoidance scale of the Odor Awareness Scale (Smeets et al., 2008). Furthermore, odor quality tends to differ with concentration (Gross-Isseroff and Lancet, 1988) such that an odorant that has shown effective priming at lower concentrations may be associated with a different odor perception at higher concentrations. Thus, we recommend that in order to achieve the presumed effects to use odors at low intensities (cf. Köster and Degel, 2000).

### SEMANTIC PRIMING

Priming via words can yield specific effects, as words would be used to pinpoint specific members and sub members of a taxonomy. Thus, the word “butterfly” could in theory be an effective prime for processing other words not just denoting insects, but specifically insects with wings. It will be clear from the above that such specific priming is unlikely to work using odors as primes. It would require not only that the odor is appropriately categorized but probably also identified by name. Knowing that individuals categorize odors in terms of, e.g., “fruity” should caution the experimenter not to use multiple fruity odors as primes. While pictures of a lemon, grapefruit and lime would possibly be easily identified by most people, this cannot be expected for the odors these fruits produce. They may be categorized as “fruity,” or “citrus.” This does not necessarily imply individuals could not discriminate between the odors at the perceptual level, but being unable to assign these odors to different categories may result in ineffective odor priming at the subcategory level.

Thus, if some form of semantic priming is intended, it is recommended to use an odor that fits a often-used category such as floral or fruity, and is a good prototype for the category (e.g., orange for citrus). Also, to ensure the priming effect was semantic/categorical there would have to be an appropriate control, for example for affective priming via odor valence. It would be good to include an odor that is equally liked or disliked but clearly does not belong to the same semantic odor category. To illustrate this we refer to a series of studies reported in Holland et al. (2005). Evidence of semantic olfactory priming was shown in a study where exposure to a citrus (cleaning agent) smell prompted subjects to express more cleaning behaviors than in the no-odor condition. Holland et al. (2005) used a Lexical Decision Task to show that a cleaning related concept had been activated through the exposure to the citrus scent. In a more applied study De Lange et al. (2012) used a similar citrus odor to show that train wagons scented with it were less littered by than unscented wagons. The activation of a cleaning related concept is held responsible for the behavioral effect of the odor prime in this study. The task in the testing phase is a behavioral one in both studies, and in addition a Lexical Decision Task in Holland et al. (2005). The authors claim that the odor activates a cleaning concept based on a past learned association of the odor with cleaning, resulting in an increased likelihood of cleaning related behavior (and faster recognition of cleaning related words in the Lexical Decision Task). However, as their studies only used one type of odor, alternative explanations related to, e.g., the valence of the odor cannot be ruled out.

In a recent study (Dijksterhuis et al., 2013), modeled after the Holland et al. (2005) study, we primed subjects with three odors of different nature. An orange odor (a citrus odor, pleasant, but with no *a priori* expected association to cleaning), a grass odor (also pleasant, but with no *a priori* association to cleaning), and a sulfur odor (unpleasant, and also not related to cleaning). The odors were presented at very low intensities in a neutral testing room, so that they were not attentively noticed. In the test phase of this study the “rusk eating task” as introduced by Holland et al. (2005) was used. The subjects were to cut and eat a rusk in a sham sensory study, and their behavior was observed to asses if and how much spontaneous cleaning actions (like wiping the table, picking up crumbs, etc.) subjects displayed under the different odor conditions. It turned out that under the sulfur condition our subjects displayed less table wiping actions than under the grass and orange odor. What this study shows is that other types of priming, than semantic priming, may be at play. The sulfur odor is unlikely to carry a semantic connotation to cleaning (more to dirt, in fact), nor do the grass and orange odors, yet they differ in the amount of cleaning behaviors they afford. We pose that the affective value of the odor can provide an alternative mechanism to explain the priming power of odors. We point out that the Dijksterhuis et al. (2013) study would have to be replicated including a no odor condition.

While on the topic of semantic odor priming it is of interest to note that Degel et al. (2001) posit that in fact, being able to verbally label an odor, seriously* interferes* with implicit priming effects. This may be due to the fact that cognitive processing of language may be disruptive to the implicit processing of odor, as the use of labels would cause a spreading of activation causing different cognitive and behavioral effects than spreading of activation solely by odor stimulation would.

#### Semantic priming via autobiographical memory

We have seen that odor priming via specific autobiographical memories can be potent and provide a gateway into semantic priming via concepts elicited by such memories. Clearly, odor priming via memory could be a very powerful application to entice consumers into buying products. The problem is that autobiographical memories are by definition personal. An odor experience that emotes one person may not do much for someone else. There are two approaches to this. One is to focus on odor experiences that are strongly linked to universally pleasant events and occasions and use these as primes to create an attraction to a product. For example, the smell of suntan lotion – often a fragrance heavy on coconut – has been reported as being associated with being on vacation in sunny locations. Thus, anecdotally, we have heard that some travel companies subtly fragrance their promotional material with coconut fragrance or provide pouches with suntan lotion along with it. This way, smelling the lotion might intensify the desire to take a vacation thus lowering the threshold for actually booking one. Likewise, parents have reported to experience feelings of melancholy and warmth when smelling the fragranced baby products (lotion, shampoo) they tended to use on their offspring, as it reminds them of nurturing their children when they were still babies. That such smells can in fact be good behavioral primes was demonstrated in an explorative study in which we first combined a novel fruity or floral odor with watching a movie in which parents interact with their babies in a loving way. In an unrelated session we later found that the smell that had been previously associated with watching the movie yielded a higher nurturing behavior score on a baby-doll than an unrelated equally pleasant smell (Smeets et al., 2010).

The second way in which learnings from the Proust effect can be used for application is by creating a memory by cleverly pairing an odorant with a certain experience so as to impart the nature of the experience onto the odorant. A subsequent encounter with that odorant would then be expected to act as prime for the experience. In their paper Degel and Köster (1999) describe an odor priming study including a learning phase. They had subjects perform a task in some rooms where an ambient odor was present. It was explicitly assessed afterward, that the subjects had not attentively perceived the odor while they were in the room. In the test phase subjects were to score the fit of the odors, now presented in jars, to environments – including the rooms they had been in – presented on photographs. A higher fit of the odor to the room the subjects had encountered the odor in was found for two out of three odors, illustrating a clear case of olfactory (repetition) priming. This cannot be attributed to some sort of a recognition effect as this would imply an explicit evaluation (recognition is an explicit function), which the authors preclude by making sure the subjects did not consciously perceive the odor in the room, with a judgment task in the test phase of the study that may be linked to the *familiarity* primary dimension of odor perception.

This result is related to the Olsson (1999) research that showed that negative priming occurred when odors that were correctly identified were proven to be processed more slowly than odors that had not been identified.

### MULTI-MODAL PRIMING

During the multiple experiments we have conducted over the past years we have found that presenting odorants in typical lab experiments did not yield the expected effects. Odorants from very different sources – rotten eggs, pizza, brownie, etc., – when asked, often gave rise to labels as “sweaty,” “computer-smell,” “rubber,” “stale-lab smell,” which was sometimes bewildering. Clearly, laboratory environments are not meaningful contexts when trying to establish an appropriate understanding of odorants and their sources. Although theoretically it is possible that odors connect up with the appropriate concepts in the brain even when subjects cannot describe them, in everyday situations we rarely ever encounter odors completely in isolation and without proper context. Thus, odors probably need help channeling to the concept of interest (Wilson and Stevenson, 2006). An obvious solution would seem to pair ambiguous odors with positive or negative labels such as “parmesan cheese” or “vomit” as Herz and von Clef (2001) did, but that would make the odor and its quality explicit thereby potentially ruining the odor priming effect. Thus, an alternative solution could be to establish cross-modality correspondences using inconspicuous combinations of olfactive and other-modality stimuli (Stevenson et al., 2012), in order to help bring out a property of the jointly presented odor such as, e.g., “softness”. Likewise, Gottfried and Dolan (2003) empirically demonstrated that semantically congruent visual information facilitated low level odor detection in congruent odor – picture pairs. Another solution is to provide context in other ways. For example if the intention is to convey the meaning of green grass and not just positive valence when presenting a green grass odor, one could put up a poster of a soccer field or have copies of soccer magazines in the waiting room to the experiment. By already making the concept accessible this way, the odor would be more likely to act as semantic prime, rather than as an affect prime, during subsequent testing. This approach is in line with the recommendations by Degel and Köster (1998) for effective odor priming:

1. the test does not supply explicit information about the stimuli,
2. the test acknowledges the nonverbal character of odor perception and memory,
3. the test allows perception of odors in a situation which is for the most part a biotic, normal everyday situation.

The latter point would explain why priming with odors in laboratories out of context is often bound to fail.

## TO CONCLUDE: A FUTURE RESEARCH AGENDA FOR EFFECTIVE ODOR PRIMING

The goal of this review was to evaluate the suitability of odors as primes. The unique properties of the sense of smell make odors both more, as well as less, suitable as primes than, e.g., visual primes (depending on the type of priming). Since most people show a natural inclination to pay more attention to visual than olfactory attributes of the environment, olfactory stimuli tend to stay outside of awareness when considering complex environments. This is especially true when they are present at low levels, where they are expected to be more evocative than at high levels. On top of this, there is the fact that the sense of smell adapts rapidly to stimulation. All these properties would lead us to conclude that environmental odors may be considered to be even more suitable to act as primes that subconsciously affect information processing and behavior than visual stimuli. Especially in relation to food, odor primes would be expected to be very powerful, as we have seen that edibility is one of the primary dimensions of odor perception. For example, when smelling an odor, people might say they like the odor, to quickly follow up by saying it is the smell of food.

Olfaction may be conceived of as a sense whose purpose, if you will, is to act as a conduit quickly channeling the olfactory input to guide approach and avoidance behavior to or away from foods, mates, predators and toxic materials. After stimulation, emotions and memory traces are rapidly evoked to facilitate such channeling in a powerful manner in the interest of survival. Where the function of olfaction, then, seems to be to discriminate at a relatively coarse level between what environmental elements either sustain or threaten survival, the visual sense acts to add detail, and subject what is in the environment to more fine-grained analysis. As a result, olfactory primes are prone to do well when priming emotionally loaded cognitive processes and behaviors, but not so well when the processing requires analyses with high levels of detail. From these features, it may be inferred that odors make for great behavioral and goal primes, but presumably not for great semantic primes. Construal priming much depends on how the odor prime is interpreted, which cannot always be reliably predicted. Adding subtle contextual features to help channel the prime to the intended concept, or create an emotional experience around the odor prime, that will result in an emotional memory that, once associated with the prime, will assist channeling the prime to concepts encoded in memory.

To find effective odor primes for applied purposes, we advocate the following research agenda. Firstly, to investigate whether effective odor primes are successful because the underlying effect is one of affective priming versus semantic priming, a supposedly semantic odor prime should always be compared to another odor prime, matched for valence but unrelated to the intended semantic category. If it is found that other similarly valenced odors are equally effective, priming can be extended to include many other odor primes than only the one believed to have specific meaning.

Furthermore, the role of odorant concentration (and its perceptual pendant odor intensity) is very important. With increasing concentrations, odorants become detectable (“There is something.”), then recognizable in terms of general quality or category (“It is fruity.”), then potentially identifiable (“It is orange.”). Now on the one hand identifiability might act as an aid to semantically channeling the odorant input to a concept, thus making it a semantic prime. On the other hand, as soon as the odor is strong people become aware, then, priming is unlikely to take place. This is in line with Loersch and Payne (2011) observations that extreme primes are less likely to have the expected priming effects, but instead, may even lead to contradictory outcomes. In addition, as soon as an odor is verbally labeled, cognitive processing is no longer implicit or automatic. Likewise, sensory profiles of odors tend to change with increasing concentrations of odorants. While the odorant composition is still the same, the mental representation associated with it, is not. Thus, research systematically investigating effects of changes in concentration of the odorant leading to the odor prime on the efficiency of the prime would help us find the most effective concentrations for priming. Because of a misunderstanding that odors must be strong and clearly perceivable, many intended odor primes are probably not as effective as they could be.

Finally, we expect that odors can become semantic primes with a little help from other-modality friends. After all, in accordance with the situated inference model, the nature of the prime depends on the situation (Loersch and Payne, 2011). Again, subtlety is king. Including some not-too-obvious cues can help give meaning to an odor prime while adding to the effect of the other-modality cue thus making it more effective (cf. Degel and Köster, 1998). Moreover, by linking the odor to an emotional experience around relevant concepts, it may be expected that the experience is encoded as a memory, encoding the odor along with it thus increasing the likelihood that the odor will act as semantic prime to these concepts on a subsequent encounter.

Olfactory priming exists. That is: the literature provides much support for the notion that odor priming in terms of an “effect” (i.e., odors activating related representations, all linked together in a conceptual network of mental representations) is a reality. However, the specific psychological and physiological processes responsible for the effects still need to be elucidated. The mechanism underlying priming may not be the same for visual and odor priming. In discussing matters of olfactory perception we have to beware not to mix the concepts odorant and odor, as there is not a one-to-one relationship between the two. There are several specific properties of the sense of smell that need closer scrutiny. Some properties make the olfactory sense a good or a not-so-good sense for priming depending on the type of priming. The nature of the odor, its intensity, and the context in which an odorant is presented has a great influence on the specific priming effect that will be experienced. Finally, the extent to which olfactory priming can be conceived of as semantic priming as opposed to affective priming providing the more parsimonious explanation, needs to be further explored.

We have taken first steps in setting an applied research agenda for olfactory priming, listing some topics of both theoretical and practical relevance. If we do it right, olfactory priming holds great promise.

## AUTHOR CONTRIBUTIONS

The first author was invited to submit this article and contributed to the idea and main structure of the paper. The second author contributed specific texts and both authors subsequently shaped the paper.

## Conflict of Interest Statement

The authors declare that the research was conducted in the absence of any commercial or financial relationships that could be construed as a potential conflict of interest.
